# Treatment of renal leiomyosarcoma with right atrial tumor embolus without cardiopulmonary bypass: a case report

**DOI:** 10.3389/fonc.2024.1385073

**Published:** 2024-05-10

**Authors:** Yuanpeng Duan, Chengwei Bi, Guoying Zhang, Yapeng Xing, Yang Qin, Bin Zhao

**Affiliations:** Department of Urology, the Third Affiliated Hospital of Kunming Medical University, Kunming, Yunnan, China

**Keywords:** primary renal leiomyosarcoma, inferior vena cava tumor thrombolus, artificial vascular replacement, cardiopulmonary bypass, right atrial tumor embolus

## Abstract

**Objective:**

To investigate the clinical manifestations, imaging and pathological features, treatment methods and prognosis of primary leiomyosarcoma of kidney, and the choice of treatment with tumor thrombus.

**Methods:**

The clinical data of a patient with primary renal leiomyosarcoma complicated with inferior vena cava and right atrial tumor thrombus were retrospectively analyzed. Radical resection of right kidney without cardiopulmonary bypass and removal of inferior vena cava and right atrial tumor thrombus were performed. Adjuvant intravenous chemotherapy was given according to the results, and follow-up observation was made.

**Results:**

Postoperative pathological findings were: leiomyosarcoma (right renal tumor), the size of the mass was about 12.1 cm, and no cancer was found at the incision end of the right ureter.

**Conclusion:**

Primary leiomyosarcoma of kidney is rare in clinical practice, and complication of right atrial tumor embolus is even rarer. The disease has high malignant degree and poor prognosis. The clinical manifestations and imaging examinations were non-specific, and pathological diagnosis was the gold standard. Radical surgical resection is the main treatment method at present, and it provides experience for the treatment of grade IV tumor thrombus without cardiopulmonary bypass.

## 1 Clinical data

**Figure 1:**
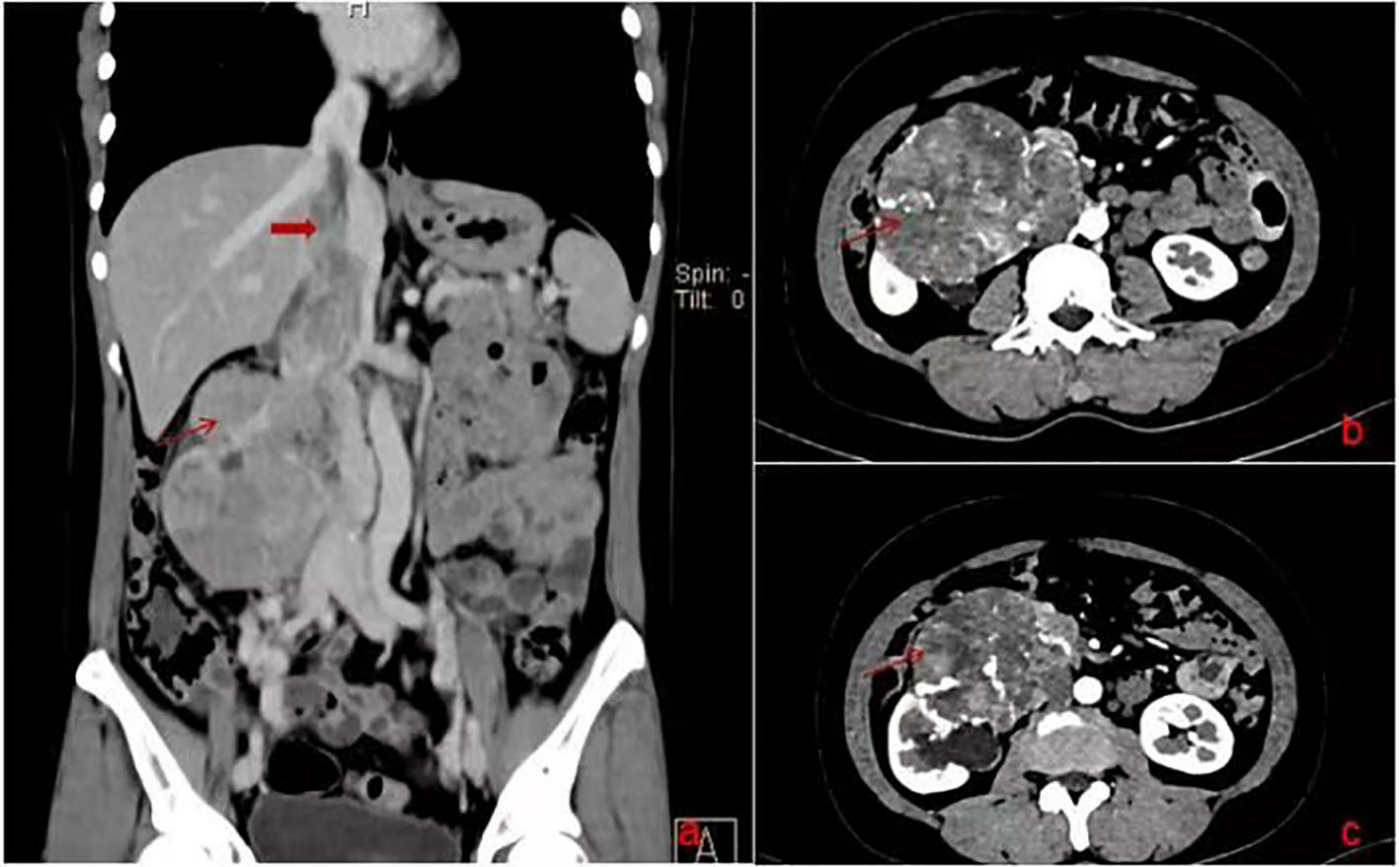
**(A)** is preoperative coronal CT. **(B)** and C is preoperative horizontal CT.

**Figure 2:**
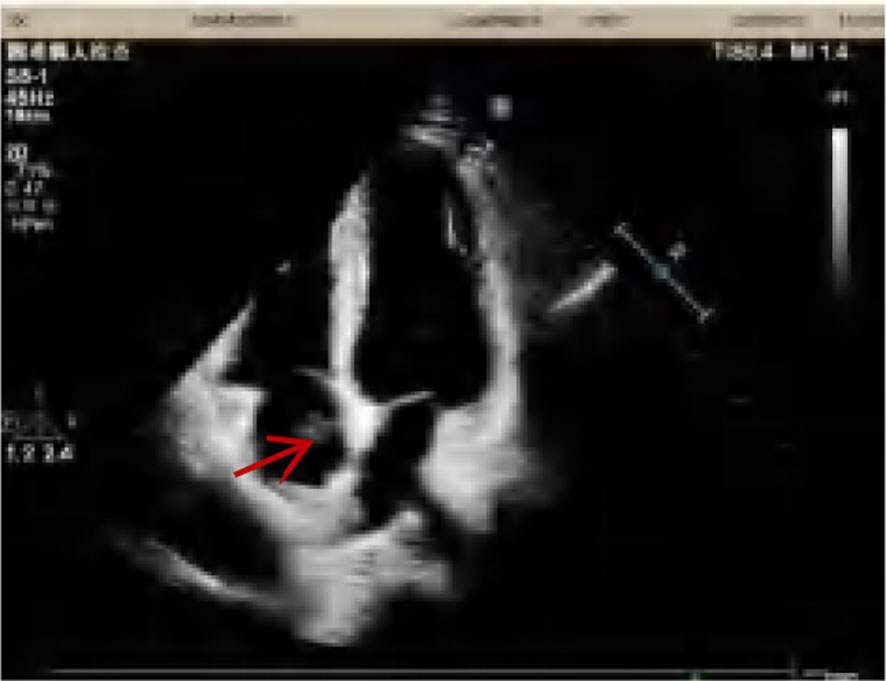
Preoperative cardiac ultrasound, the arrow points to a cancer embolus.

A 47-year-old female patient was admitted to our hospital with intermittent right abdominal pain without obvious inducement 6 months ago, which could be relieved by itself and was not noticed. The abdominal pain worsened 1 week ago, and the examination of other hospitals indicated retroperitoneal tumor, so she was admitted to our hospital for further diagnosis and treatment. Physical examination: abdominal wall was soft, no abdominal mass was touched, no tenderness, rebound pain, no percussion pain in both kidney areas. Improved enhanced CT and MRI showed that right renal carcinoma, about 12.1*8.5*11.8cm in size, invaded the renal pelvis, and invaded the inferior vena cava through the renal vein, forming a grade IV cancer thrombus (**Figure 1**). Echocardiography showed that a 16*13mm space could be detected in the right atrium, which seemed to have pedicle and inferior vena cava continuation, and could reach the tricuspid valve opening with blood flow (**Figure 2**). Diagnosis under consideration: renal malignancy with grade IV thrombus (Mayo grade). Multiple departments such as abdominal surgery, thoracic surgery, imaging, anesthesiology, Interventional surgery, and medical oncology were invited to conduct multidisciplinary preoperative discussions in our hospital. The results of the discussion agreed to assist the operation of descending right kidney tumor resection and removal of vena cava and right atrial tumor thrombus. Prepare for thoracotomy thrombectomy. Low molecular weight heparin was used for continuous anticoagulation for 3 days before surgery. CT and cardiac ultrasound were re-examined 1 day before surgery. No significant changes were observed in renal tumor and inferior vena cava tumor thrombolus, and the tumor thrombolus in the right atrium was reduced to about 15*10mm in size. 

**Figure 3:**
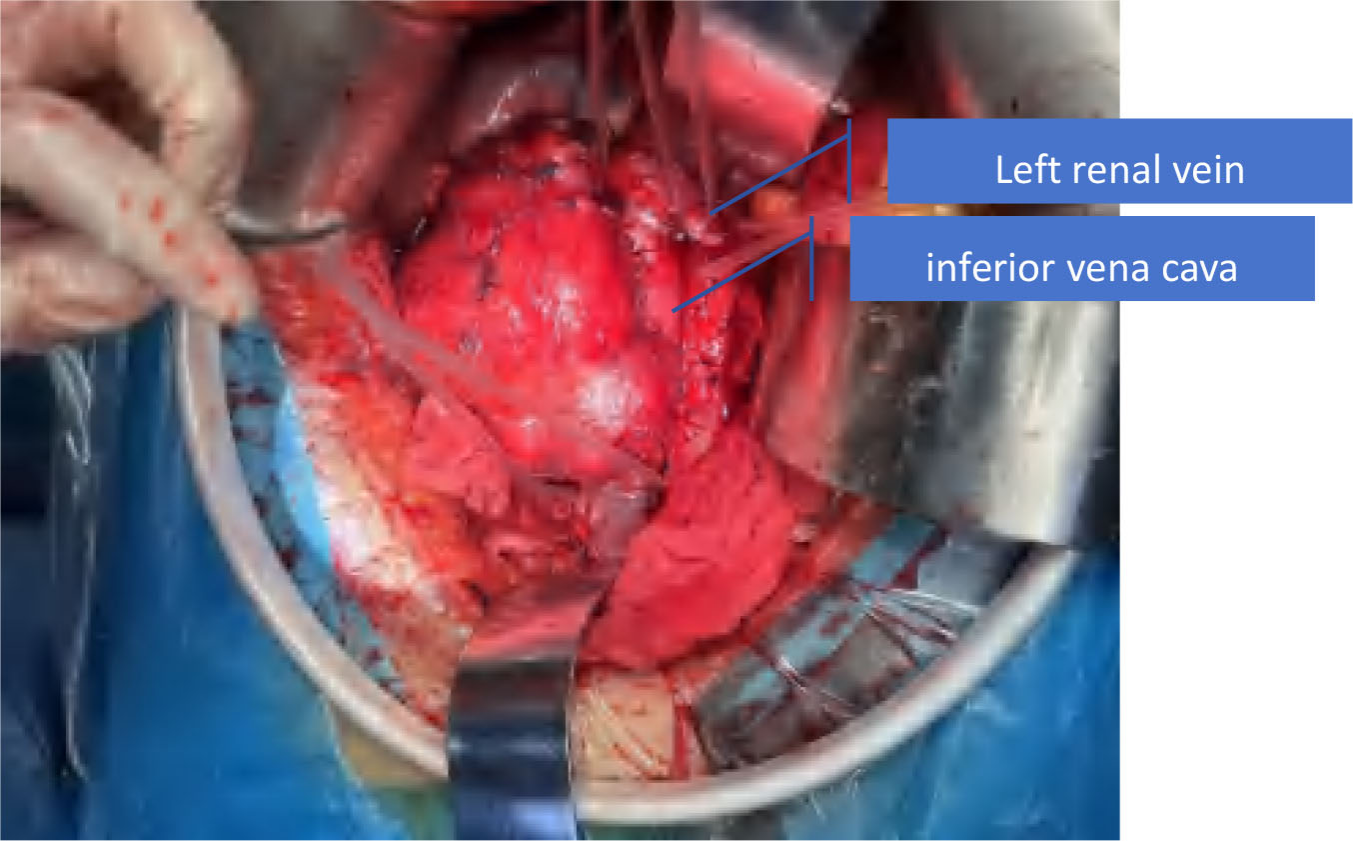
Tumor and surrounding organs.

**Figure 4:**
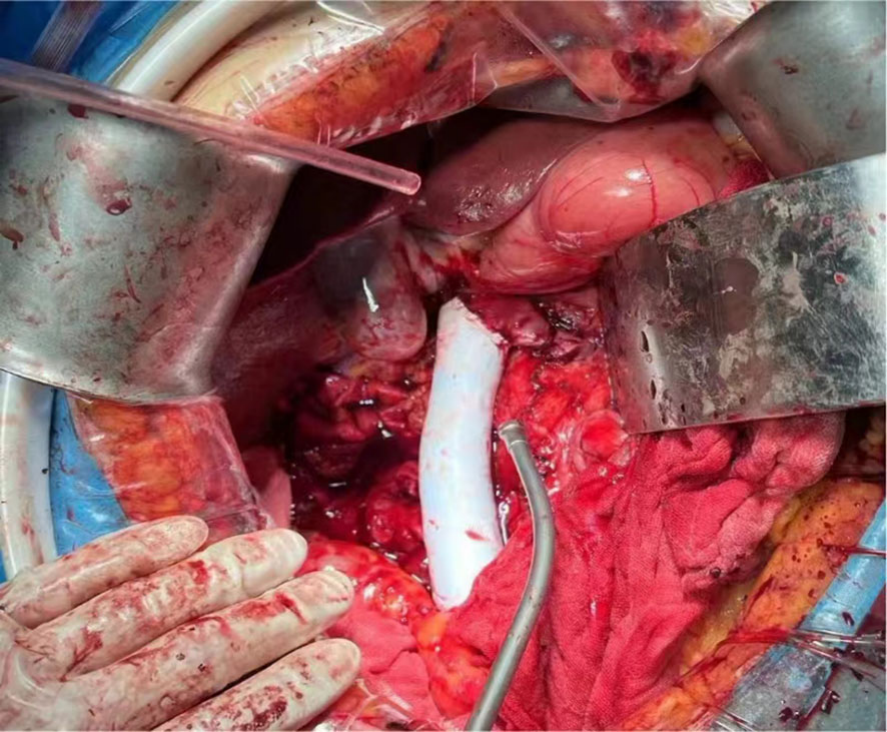
An artificial vascular replacement has been performed.

**Figure 5:**
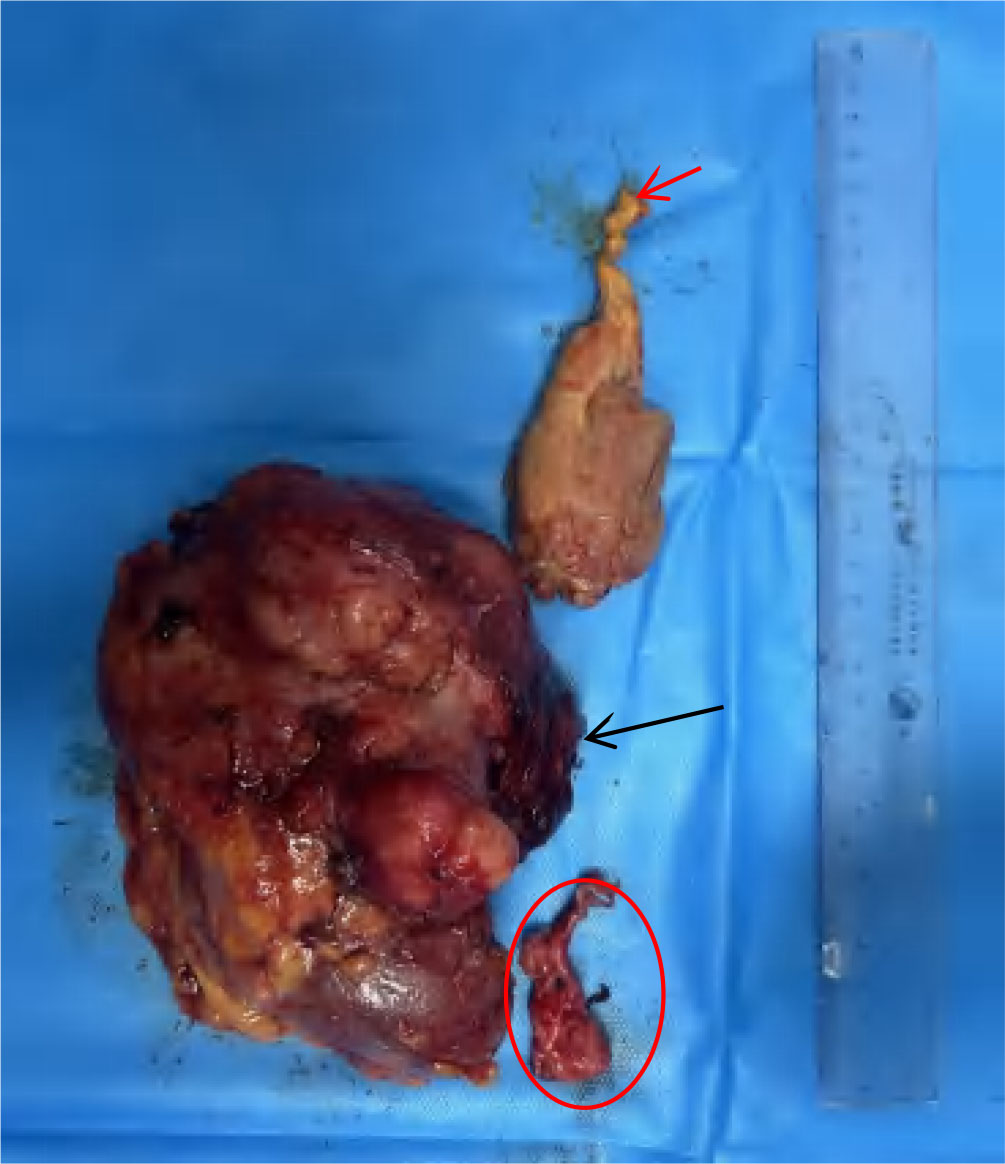
Postoperative, the red arrow points to right atrial tumor thrombus, the black arrow points to right renal and tumor, the ellipse is the wall of the inferior vena cava invaded by the tumor.

The patient underwent “huge right kidney tumor resection + removal of vena cava and right atrial tumor embolus + inferior vena cava artificial vascular replacement” on September 19, 2023. Median abdominal incision was performed (Prepare for thoracotomy). During the operation, the tumor was located on the right side and front of the inferior vena cava (IVC), showing invasive growth, surrounding and invading about 2/3 of the diameter of the IVC, invading the IVC wall length of about 8cm, and the IVC vascular wall could not be separated. The right renal vein was completely enveloped by the tumor. The anteromedial tumor was closely attached to the duodenum and could be separated after sufficient dissociation. The duodenum was not damaged, and no obvious lymph node enlargement was observed around the renal portal and abdominal aorta. Free ligation of severed right renal artery, lumbar vein, ureter, and perivena cava were performed. After blocking IVC below the tumor and IVC above the left renal vein tumor without damage, the right renal tumor and the tumor invaded IVC vascular wall were completely removed. The tumor plug was removed along the vascular wall, and no fracture of the tumor plug was detected. Intracardiac ultrasound showed no intracardiac thrombosis or pulmonary artery thrombosis, and the residual IVC was insufficient for vascular repair (**Figure 3**). Therefore, Dacron fiber braided material was used to replace the defective IVC (**Figure 4**). The operation lasted for a total of 5h with 700ml bleeding and no intraoperative blood transfusion. Creatinine was 78umol/l and urea was 122mmol/l. On the first day after surgery, a small amount of liquid diet and exercise were allowed (**Figure 5**).

**Figure 6:**
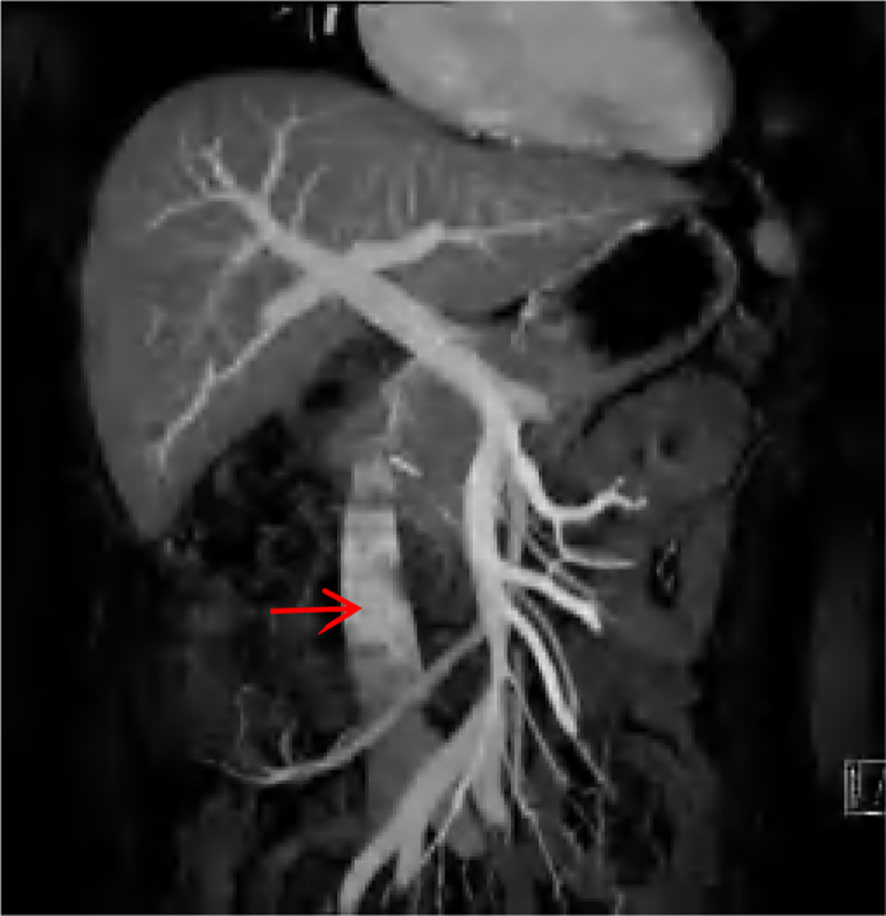
Postoperative ct, the arrow points to artificial vascular replacement.

On the fifth day after surgery, due to the increase of D2 aggregation to 22, CT angiography (CTV) was re-examined, and a small number of sheet filling defects were observed in the artificial blood vessel replacement segment (**Figure 6**). Considering thrombosis, enoxparin, 4000U, bid, subcutaneous injection was given. After discharge, rivaroxaban 20mg/d oral anticoagulant therapy was changed, CTV was regularly reviewed, and the patient recovered from the hospital on the ninth day after surgery. Postoperative examination (immunohistochemistry): Leiomyosarcoma, CK (focus +/-), Vim (+) Ki67 (+, about 30%), EMA (-), SMA (+), Desmin (focus +), Caldesmon (part +), Actin (+), HMB45 (-), S100 (-), CD34 (vascular +), MyoD1 (-), TLE1 (partial +), CD117 (-), DOG1 (-), BCL2 (+), CD99 (+), STAT6 (-), MDM2 (-), CDK4 (-), melon-A (-), CD10 (+), CYCLIND (-), SOX10 (-). After surgery, gemcitabine combined with pirarubicin was given adjuvant chemotherapy. After 3 months of follow-up, the patient had no obvious discomfort and no obvious recurrence and metastasis.

## 2 Discussion

Primary renal leiomyosarcoma is a rare type of renal malignancies originating in the mesenchymal tissue and often originating in the renal venous smooth muscle, accounting for about 0.12% of all renal malignancies (1). The disease tends to occur in middle-aged and elderly people, with an average age of 58.5 years, and is more common in the right kidney, with a higher prevalence rate in females than in males, with a ratio of 3:2 (2). Mesenchymal leiomyosarcoma is often large due to the lack of tissue barrier, and has a high potential for recurrence and metastasis. The main clinical manifestations are mass, pain and gross hematuria.

Compared with other urinary malignancies, primary renal leiomyosarcoma has a poor prognosis. Complete resection of the tumor is positively correlated with overall survival, and radical resection is the preferred option for patients (3). There is no difference in survival between advanced renal LMS patients receiving chemotherapy and those who do not receive chemotherapy (4). Primary renal leiomyosarcoma and renal cell carcinoma are difficult to distinguish in preoperative diagnosis, both of them have similar clinical symptoms and imaging findings, and postoperative pathological results are the qualitative gold standard. Pathologically distinguishable from sarcomatoid renal cell carcinoma, leiomyosarcoma has a monotypic nucleus rather than the multitypic cells of sarcomatoid carcinoma. In the absence of smooth muscle markers, sarcomatoid renal cell carcinoma is diagnosed when cytokeratin is positive. If leiomyosarcoma expresses cytokeratin or epithelial membrane antigen (EMA), Desmin staining can be used to identify leiomyosarcoma, as they are positive in leiomyosarcoma but not positive in sarcomatoid carcinoma (5).

Renal malignancies may extend into the vein to form a tumor embolus and may further extend into the inferior vena cava or even the right atrium. At present, the widely accepted classification method is the inferior vena cava tumor classification method of Mayo Medical Center (6). Specifically: Grade 0, the tumor plug is confined to the renal vein; In grade I, the tumor plug extended into the inferior vena cava, and the apex was less than 2 cm from the opening of the renal vein. Grade II: the distance between the top of the tumor plug and the opening of the renal vein was >2 cm, but lower than the level of the hepatic vein; In grade III, the tumor was extended to the level of the intrahepatic inferior vena cava, but lower than the level of the diaphragm. In grade IV, the tumor stops in the inferior vena cava and extends above the level of the diaphragm.

In the past, it was considered that renal malignant tumors with inferior vena cava thrombolus had a very poor prognosis and should not be treated with active surgery. However, recent data analysis showed that inferior vena cava thrombolus had no significant prognostic significance (7), and many patients could achieve long-term survival after complete resection of tumor and thrombolus. Therefore, surgical removal of the tumor and its thrombolus is the only chance of cure for most patients. For renal vein thrombus, because the tumor thrombus has not invaded the inferior vena cava, it is not necessary to block the inferior vena cava during operation, and only clamp the renal vein at the proximal cardiac end of the tumor thrombus. The Grade II tumor plug is pulled back to the renal vein by laparoscopy or robot-assisted laparoscopy, thus reducing the difficulty of the operation. The operation method of the grade I tumor plug is also suitable. However, if the tumor plug is too extensive, it is difficult to pull back to the renal vein, and then surgery such as vena cava incision is needed to remove the tumor plug (8, 9). The tumor plug of grade III was removed by cutting the vena cava after clamping the inferior vena cava and renal vein. Thoraco-abdominal incision was also used for grade IV thrombus surgery, similar to grade III thrombus surgery. It should be noted that the large blood vessels of the heart should be fully exposed for cardiopulmonary bypass. If the thrombus extends to the right atrium, the right atrium should be opened for thrombus removal. When the tumor thrombus is at a high position or even at the level of the right atrium, cardiopulmonary bypass technology, cryogenic circulatory stop technology or veno-right atrium natural bypass technology should be applied to reduce intraoperative bleeding. Surgeons can remove the tumor thrombus or replace blood vessels in the “blood-free state” to prevent the spread of cancer cells and the loss of tumor thrombus, and improve surgical safety (10, 11). Studies have also shown that satisfactory surgical effects can be achieved without the use of circulation bypass (12).

For traditional grade IV tumor embolism, thoracoabdominal incision should be considered, and cardiopulmonary bypass should be used to assist, otherwise, embolism may be caused by cancer embolism, and even acute pulmonary embolism or other organ embolism in severe patients. This operation completely “pulls” out the tumor plug in the inferior vena cava and the right atrium from the inferior vena cava incision, without thoracotomy, reducing trauma and avoiding the aid of extracorporeal circulation, which can provide ideas for this kind of surgery, but the risk is extremely high.

## Data availability statement

The original contributions presented in the study are included in the article/supplementary material. Further inquiries can be directed to the corresponding authors.

## Ethics statement

The studies involving humans were approved by Department of Urology, the Third Affiliated Hospital of Kunming Medical University. The studies were conducted in accordance with the local legislation and institutional requirements. Written informed consent for participation was not required from the participants or the participants’ legal guardians/next of kin in accordance with the national legislation and institutional requirements. The manuscript presents research on animals that do not require ethical approval for their study. Written informed consent was obtained from the individual(s) for the publication of any potentially identifiable images or data included in this article.

## Author contributions

YD: Writing – original draft. CB: Data curation, Methodology, Writing – original draft. GZ: Data curation, Methodology, Writing – review & editing. YX: Data curation, Methodology, Writing – review & editing. YQ: Data curation, Methodology, Writing – review & editing. BZ: Writing – review & editing.
